# Association Between Depression and Peripheral Artery Disease: Insights From the Heart and Soul Study

**DOI:** 10.1161/JAHA.112.002667

**Published:** 2012-08-24

**Authors:** S Marlene Grenon, Jade Hiramoto, Kim G. Smolderen, Eric Vittinghoff, Mary A. Whooley, Beth E. Cohen

**Affiliations:** 1 Department of Surgery, University of California San Francisco, San Francisco, CA (S.M.G., J.H.); 2 Department of Medicine, University of California San Francisco, San Francisco, CA (M.A.W., B.E.C.); 3 Department of Epidemiology and Biostatistics, University of California San Francisco, San Francisco, CA (E.V., M.A.W.); 4 Department of Surgery, Veterans Affairs Medical Center, San Francisco, CA (S.M.G.); 5 Department of Medicine, Veterans Affairs Medical Center, San Francisco, CA (M.A.W., B.E.C.); 6 Saint Luke's Mid America Heart Institute, Kansas City, MO (K.G.S.); 7 Center of Research on Psychology in Somatic Diseases, Tilburg University, The Netherlands (K.G.S.)

**Keywords:** depression, peripheral artery disease, risk factors

## Abstract

**Background:**

Depression is known to increase the risk of coronary artery disease, but few studies have evaluated the association between depression and peripheral artery disease (PAD). We examined the association of depression with PAD and evaluated potential mediators of this association.

**Methods and Results:**

We used data from the Heart and Soul Study, a prospective cohort of 1024 men and women with coronary artery disease recruited in 2000–2002 and followed for a mean of 7.2±2.6 years. Depressive symptoms were assessed with the validated 9-item Patient Health Questionnaire. Prevalent PAD at baseline was determined by self-report. Prospective PAD events were adjudicated on the basis of review of medical records. We used logistic regression and Cox proportional-hazards models to estimate the independent associations of depressive symptoms with prevalent PAD and subsequent PAD events. At baseline, 199 patients (19%) had depressive symptoms (Patient Health Questionnaire ≥10). Prevalent PAD was reported by 12% of patients with depression and 7% of those without depression (base model adjusted for age and sex: odds ratio 1.79, 95% confidence interval 1.06–3.04, *P*=0.03; full model adjusted for comorbidities, medications, PAD risk factors, inflammation, and health behaviors: odds ratio 1.59, 95% confidence interval 0.90–2.83, *P*=0.11). During follow-up, PAD events occurred in 7% of patients with depression and 5% of those without depression (base model adjusted for age and sex: hazard ratio 2.09, 95% confidence interval 1.09–4.00, *P*=0.03; full model adjusted for comorbidities, medications, PAD risk factors, inflammation, and health behaviors: hazard ratio 1.33, 95% confidence interval 0.65–2.71, *P*=0.44). Factors explaining >5% of the association between depression and incident PAD events included race/ethnicity, diabetes, congestive heart failure, high-density lipoprotein, triglyceride levels, serum creatinine, inflammation, smoking, and levels of physical activity.

**Conclusions:**

Depressive symptoms were associated with a greater risk of PAD. Because the association was explained partly by modifiable risk factors, our findings suggest that more aggressive treatment of these risk factors could reduce the excess risk of PAD associated with depression. **(*J Am Heart Assoc*. 2012;1:e002667 doi: 10.1161/JAHA.112.002667.)**

## Introduction

Peripheral artery disease (PAD) and coronary artery disease (CAD) share several biological and behavioral risk factors.^1–13^ Depression has emerged as a key risk factor for the development and progression of CAD, but far less is known about the association between depression and PAD outcomes.^3,14–18^ If depression is a risk factor for the incidence and progression of PAD, it could be an important modifiable target for interventions to possibly improve outcomes of these patients.

Recent reports have suggested an association between depression and PAD, but none have evaluated potential mechanisms for this association. In the Atherosclerosis Risk in Communities Study, depression was associated with greater incident PAD.^19^ In patients with existing PAD, prior studies have found that those with depression have worse functional outcomes, greater need for revascularization, poorer quality-of-life outcomes after revascularization, and higher risk for adverse events after revascularization.^20–24^ However, the factors mediating the possible effects of depression on PAD prognosis are unknown. In addition, the association of depression and PAD in patients at high risk for adverse outcomes (ie, with a history of CAD) has not been evaluated. Finally, prospective data on the effects of depression on PAD are scarce.

Because patients with CAD are at especially high risk for PAD,^25–32^ we sought to determine whether depressive symptoms were associated with having comorbid PAD as well as with the occurrence of future PAD events in a contemporary cohort of patients with CAD, and we sought to explore pathophysiological mechanisms of such an association.

## Methods

### Study Population

The Heart and Soul Study was designed to determine how psychological disorders lead to cardiovascular events in outpatients with stable CAD. Detailed methods have been described previously.^33^ Participants were recruited from 2 Departments of Veterans Affairs medical centers (San Francisco Veterans Affairs Medical Center and the Veterans Affairs Palo Alto Health Care System), 1 university medical center (University of California, San Francisco), and 9 public health clinics in the Community Health Network of San Francisco. Patients were eligible to participate in the study if they met ≥1 of the following conditions: a history of myocardial infarction, angiographic evidence of ≥50% stenosis in ≥1 coronary vessels, previous evidence of exercise-induced ischemia during treadmill use or nuclear testing, or a history of coronary revascularization. Between September 11, 2000, and December 20, 2002, a total of 1024 participants were enrolled. Participants were followed up for 7.2±2.6 years (mean ± standard deviation [SD]).

All participants completed a baseline examination that included an interview, fasting venous blood sample collection, a standardized medical history questionnaire, echocardiography, exercise treadmill testing, 24-hour ambulatory electrocardiography, and a 24-hour urine collection. Of the 1024 participants who completed the baseline examination, 1018 (>99%) had follow-up information on PAD events. The protocol was approved by the appropriate institutional review boards, and all participants provided written informed consent for participation in the study.

### Predictor: Depressive Symptoms

Depressive symptoms were assessed with the validated 9-item Patient Health Questionnaire.^34^ The Patient Health Questionnaire provides a dichotomous measure of depressive symptoms that is based on a score ≥10. A score ≥10 has a sensitivity of 88% and a specificity of 88% for major depressive disorders.^35^ The Patient Health Questionnaire-9 has been used successfully in patients with PAD previously.^22^

### Outcome: PAD

Prevalent PAD at baseline was determined by the patient's self-report of having received a prior diagnosis from a physician or a nurse. The research participant was asked, “Has a doctor or nurse ever told you that you have peripheral vascular disease?” Participants were followed up by telephone annually to assess for PAD events. For all potential events, medical records were collected and reviewed by 2 independent physician adjudicators, with review by a third physician to resolve any disagreements. Incident PAD events were identified on the basis of any of the following: final diagnosis by a physician during hospitalization (n=56); ultrasonographically or angiographically demonstrated obstruction or ulcerated plaque (>50% of diameter or >75% of cross-sectional area) of the iliac arteries or below (n=40); surgery, angioplasty, or thrombolysis for PAD (n=40); or exertional leg pain relieved by rest (n=23). All except 2 diagnoses of “exertional leg pain relieved by rest” were also positive for an ultrasonographic or angiographic diagnosis of PAD or a PAD-related procedure. We evaluated the cross-sectional association between depressive symptoms and self-reported diagnosis of PAD, as well as the longitudinal association between depressive symptoms and incident PAD.

### Patient Characteristics

Age, sex, race/ethnicity, education level, and medical history were determined by self-report questionnaire. Height and weight were measured by a standardized protocol, with body mass index calculated as weight in kilograms divided by height in meters squared. Participants were instructed to bring their medication bottles to their enrollment visit, and study personnel recorded all current medications. Medications were categorized by using Epocrates Rx (Epocrates Inc, San Mateo, CA).

### Potential Biological Mediators

Fasting blood samples were obtained during the morning of the enrollment visit. Levels of high-sensitivity C-reactive protein (CRP), interleukin (IL)-6, and tumor necrosis factor-α were determined from plasma and serum samples. High-sensitivity CRP levels were measured with the Integra assay (Roche, Indianapolis, IN) in the first 229 participants and (because of a change at the laboratory) with the Extended Range assay (Beckman Coulter Ireland, Inc, Galway, Ireland) in the remaining samples. Prior testing demonstrated high correlation of these 2 methods.^17^ We used the R&D Systems (Minneapolis, MN) Quantikine HS IL-6 immunoassay to determine the concentration of IL-6. We used the Human Serum Adipokine Panel B LINCOplex Kit (Linco Research, Inc, St. Charles, MO) to measure tumor necrosis factor-α. Low- and high-density lipoprotein cholesterol levels were measured from fasting venous blood samples at baseline.

### Potential Behavioral Mediators

Smoking (never, former, or current) was determined by self-report questionnaire. Alcohol use was evaluated with the validated AUDIT-C questionnaire.^36^ To assess medication adherence, participants were asked, “In the past month, how often did you take your medications as the doctor prescribed?” Possible responses were “all of the time (100%),” “nearly all of the time (90%),” “most of the time (75%),” “about half the time (50%),” or “less than half the time (≤50%).” We defined medication nonadherence as taking prescribed medications ≤75% of the time.^37^ To assess physical activity, the participants were asked, “Which of the following statements best describes how physically active you have been during the past month—that is, done activities such as 15 to 20 minutes of brisk walking, swimming, general conditioning, or recreational sports?” Participants chose 1 of the following 6 categories: not at all active, a little active (1 to 2 times per month), fairly active (3 to 4 times per month), quite active (1 to 2 times per week), very active (3 to 4 times per week), or extremely active (≥5 times per week). Participants who reported that they were not at all or a little active were considered physically inactive. Self-report has been shown to be a reliable, valid, and accurate method of assessing physical activity.^38–41^ In particular, single-response items have demonstrated excellent construct validity.^40–42^

### Statistical Analysis

Differences in baseline characteristics by depression status were evaluated with Student *t* tests for continuous variables and χ^2^ tests for categorical variables (no variables had >4% missing data). We used logistic models to assess the association of depressive symptoms with prevalent PAD at baseline and Cox proportional-hazards models to assess the association of depressive symptoms with subsequent PAD events. We verified the log-linearity assumption for continuous variables by checking for improvement in fit with the use of restricted cubic spline transformations of the predictor. Following convention, we log-transformed covariates with severely right-skewed distributions, including CRP, IL-6, and triglycerides. To assess the extent to which the simple age-adjusted association of depression with PAD might be explained by confounders, such as demographic variables, comorbidities, and traditional PAD risk factors, as well as potential mediators, such as inflammatory markers and health behaviors, the variables were added one at a time to the age-adjusted model, according to a previously described algorithm.^33^ These results were summarized by the percent change in the coefficient for depression after addition of the covariate, conventionally known as percent treatment effect (PTE), and by 2 hypothesis tests assessing evidence for the proposed explanatory pathway: (1) for the association between depression and the covariate and (2) for the independent association of the covariate with PAD, with adjustment for depression. All variables with PTE >5% were included in subsequent multivariate models.^43^ Antidepressants were not included because they likely are a marker of more severe depression. Any patient missing the covariate of interest was excluded from the nested models. Statistical analyses were performed in Stata/SE 12 (StataCorp, College Station, TX).

## Results

A total of 199 participants (19%) had depressive symptoms (Patient Health Questionnaire ≥10) at baseline. Patients with depressive symptoms were younger and more likely to be female than were patients without depressive symptoms (Table 1). Comorbidities were more common among patients with depressive symptoms, including history of myocardial infarction, congestive heart failure, and diabetes mellitus. Patients with depressive symptoms were also less likely to be on statins. Patients with depressive symptoms had lower high-density lipoprotein, higher triglyceride, and higher serum creatinine levels. They also demonstrated a higher CRP level. Finally, behavioral risk factors were more common among patients with depressive symptoms, including current smoking, physical inactivity, medication nonadherence, and higher body mass index. The percent of age-adjusted association between depression and incident PAD events explained by different confounders and mediators is presented in Table 2.

**Table 1.** Characteristics of Patients With and Without Depressive Symptoms

General Characteristics	With Depressive Symptoms (n=199)	Without Depressive Symptoms (n=825)	*P*
Age, mean±SD, y	63±12	68±10	<0.001
Male sex, n (%)	152 (76)	688 (83)	0.02
White, n (%)	110 (55)	505 (61)	0.12
Comorbid conditions, n (%)			
Hypertension	151 (76)	572 (70)	0.06
History of myocardial infarction	121 (62)	426 (52)	0.01
Revascularization	109 (55)	493 (60)	0.25
Congestive heart failure	49 (25)	130 (16)	0.003
History of stroke	33 (17)	115 (14)	0.32
Diabetes mellitus	68 (34)	197 (24)	0.003
Medications, n (%)			
Aspirin	151 (76)	641 (78)	0.58
Angiotensin-converting enzyme inhibitor	104 (52)	420 (51)	0.73
β-Blocker	119 (60)	474 (57)	0.55
Statin	113 (57)	544 (66)	0.02
Diuretics	68 (34)	233 (28)	0.10
Antidepressant	75 (38)	113 (14)	<0.001
PAD risk factors, mean±SD			
Cholesterol, mg/dL	181±48	177±41	0.29
Low-density lipoprotein, mg/dL	105±36	104±33	0.62
High-density lipoprotein, mg/dL	44±13	46±14	0.02
Log triglycerides, mg/dL	4.9±0.7	4.7±0.6	<0.001
Systolic blood pressure, mm Hg	132±23	133±20	0.48
Diastolic blood pressure, mm Hg	75±12	75±11	0.64
Serum creatinine, mg/dL	1.3±1.1	1.1±0.5	0.002
Inflammation, mean±SD			
Log CRP, mg/L	0.89±1.33	0.67±1.30	0.04
Log IL-6, pg/mL	1.02±0.74	0.94±0.70	0.14
Log tumor necrosis factor-α, pg/mL	1.25±0.94	1.24±0.85	0.85
Health behaviors, n (%)			
Smoking			<0.001
Never	57 (29)	258 (31)	
Former	74 (37)	432 (52)	
Current	67 (34)	134 (16)	
Physically active	84 (42)	565 (69)	<0.001
Medication adherence	167 (85)	765 (93)	<0.001
Alcohol use	55 (28)	238 (29)	0.71
Body mass index, mean±SD, kg/m^2^	29±6	28±5	0.02

SD indicates standard deviation; PAD, peripheral artery disease; CRP, C-reactive protein; IL, interleukin.

**Table 2.** Adjusted Relative Hazards for the Association Between Depression and PAD,* With Percent of the Effect Explained (PTE) by Covariate

Covariate	Hazard Ratio	95% CI	*P*†	*P*‡	PTE
Male	1.77	1.02–3.08	0.11	0.14	−4.2
White	1.59	0.91–2.79	0.02	0.10	15.5
Comorbid conditions					
Hypertension	1.69	0.97–2.94	0.03	0.02	4.8
History of myocardial infarction	1.75	1.00–3.04	0.02	0.30	4.2
Revascularization	1.78	1.03–3.09	0.49	0.003	−3.9
Congestive heart failure	1.50	0.85–2.62	0.004	<0.001	26.4
History of stroke	1.71	0.98–2.96	0.09	0.13	4.1
Diabetes mellitus	1.68	0.97–2.93	0.01	0.15	5.5
Medications					
Aspirin	1.75	1.01–3.05	0.71	0.04	−2.3
Angiotensin-converting enzyme inhibitor	1.73	0.99–3.00	0.48	0.14	0.8
β-Blocker	1.71	0.98–2.97	0.44	0.05	2.6
Statin	1.81	1.04–3.14	0.08	0.02	−7.6
Diuretic	1.66	0.95–2.88	0.03	0.02	8
PAD risk factors					
Cholesterol (mg/dL)	1.74	1.00–3.02	0.81	0.52	−0.9
Low-density lipoprotein (mg/dL)	1.36	0.74–2.50	0.93	0.97	−0.1
High-density lipoprotein (mg/dL)	1.65	0.95–2.87	0.05	0.01	8.6
Log triglycerides (mg/dL)	1.65	0.95–2.88	0.02	0.008	8
Systolic blood pressure (mm Hg)	1.70	0.96–2.99	0.93	0.04	−2.7
Diastolic blood pressure (mm Hg)	1.68	0.95–2.96	0.44	0.68	−1.1
Serum creatinine (mg/dL)	1.59	0.90–2.80	0.004	0.003	15.9
Biomarkers					
Log CRP (mg/L)	1.89	1.08–3.31	0.04	0.30	3.8
Log IL-6 (pg/mL)	1.75	1.00–3.07	0.01	<0.0001	14.3
Log tumor necrosis factor-α (pg/mL)	1.97	1.13–3.44	0.66	<0.0001	−3.2
Health behaviors					
Smoking	1.57	0.90–2.74	0.0008	0.0002	18.4
Physically inactivity	1.63	0.92–2.88	<0.0001	0.22	14.9
Medication adherence	1.76	1.01–3.06	0.002	0.84	−0.9
Alcohol use	1.71	0.98–2.97	0.55	0.21	1.6
Body mass index	1.76	1.01–3.06	0.13	0.44	−2.9

In the base age-adjusted Cox model for incident PAD, the relative hazard for depression was 1.73 (95% CI 1.0–3.01). The percent of the age-adjusted association explained (PTE) was calculated as the relative change in the regression coefficient for depression after the covariate was added to the age-adjusted model. PAD, peripheral artery disease.

* Positive numbers indicate that adjustment for the covariate reduces the effect size of depression on PAD; negative numbers indicate that adjustment for the covariate strengthens the effect size.

† *P* for the association of depression with the covariate.

‡ *P* for the association of the covariate with incident PAD, with adjustment for depression.

Prevalent PAD was reported by 12% (24/199) of patients with depressive symptoms and 7% (60/825) of participants without depressive symptoms (base model adjusted for age and sex: odds ratio 1.79, 95% confidence interval [CI] 1.06–3.04, *P*=0.03). This association remained strong after adjustment for individual clusters, including comorbid conditions, PAD risk factors, inflammation, and health behaviors. However, it was no longer significant after adjustment for all mediators together (Table 3; full model: odds ratio 1.59, 95% CI 0.90–2.83, *P*=0.11; refer to Table 4 for full model details). Together, these factors explained 20.3% of the age-adjusted association of depression with prevalent PAD.

**Table 3.** Association Between Depression and Self-Reported PAD at Baseline With Sequential Adjustment for Potential Confounders and Mediators*

Variable	Odds Ratio (95% CI)	*P*
Adjusted for age and sex only	1.79 (1.06–3.04)	0.03
Adjusted for age, sex, and race/ethnicity	1.88 (1.10–3.20)	0.02
Adjusted age, sex, race/ethnicity comorbid conditions, and medication use	1.61 (0.93–2.78)	0.09
Adjusted for age, sex, race/ethnicity and PAD risk factors	1.81 (1.06–3.10)	0.03
Adjusted for age, sex, race/ethnicity and inflammation	1.77 (1.04–3.03)	0.04
Adjusted for age, sex, race/ethnicity and health behaviors	1.79 (1.03–3.11)	0.04
Adjusted for age, sex, race/ethnicity comorbid conditions, medication use, PAD risk factors, inflammation, and health behaviors	1.59 (0.90–2.83)	0.11

Adjusted associations between depression and history of PAD at baseline were estimated by using logistic models with sequential addition of candidate mediators. PAD, peripheral artery disease.

* Included all variables that changed the effect size between depression and PAD by >5%: demographics (age, race), comorbid conditions and medication use (history of congestive heart failure, diabetes, statins and diuretics), PAD risk factors (high-density lipoprotein, triglycerides, serum creatinine), inflammation (log IL-6), and health behaviors (smoking, physical inactivity).

**Table 4.** Full Model for Self-Reported PAD at Baseline

Variable	Odds Ratio (95% CI)	*P*
Depression	1.59 (0.90–2.83)	0.11
Age (per 5 y)	0.99 (0.88–1.12)	0.93
Male	0.83 (0.43–1.60)	0.58
Race/ethnicity		
White	1.00 (ref)	Ref
Hispanic	0.48 (0.16–1.38)	0.17
Asian	0.92 (0.41–2.04)	0.83
African American	0.74 (0.36–1.51)	0.40
Other	0.23 (0.03–1.79)	0.16
Congestive heart failure	2.06 (1.17–3.60)	0.012
Diabetes	1.63 (0.96–2.74)	0.07
Statins	0.75 (0.45–1.23)	0.25
Diuretics	1.09 (0.63–1.88)	0.76
High-density lipoprotein (per 5-mg/dL decrease)	1.04 (0.94–1.15)	0.42
Triglyceride (per natural log increase)	1.03 (0.68–1.55)	0.90
Creatinine (per 0.1 mg/dL)	0.98 (0.95–1.02)	0.45
IL-6 (per natural log increase)	1.43 (0.98–2.08)	0.07
Smoking		
Never	1.00 (ref)	Ref
Former	1.02 (0.57–1.82)	0.94
Current	1.09 (0.52–2.26)	0.82
Physical inactivity	1.03 (0.89–1.20)	0.69

Adjusted associations between risk factors including depression and history of PAD at baseline were estimated by using logistic model. PAD, peripheral artery disease.

Figure 1 demonstrates incident PAD events by depression status. Significantly more PAD events occurred in patients with depressive symptoms than in those without depressive symptoms (*P*=0.05). After exclusion of patients with a self-reported history of PAD, incident PAD events occurred in 7% (13/175) of patients with depressive symptoms and 5% (37/759) of patients without depressive symptoms (base model adjusted for age and sex: hazard ratio 2.09, 95% CI 1.09–4.00, *P*=0.03). Again, this association was no longer significant in the full model after adjustment for comorbidities, PAD risk factors, inflammation, and health behaviors (Table 5; full model hazard ratio: 1.33, 95% CI 0.65–2.71, *P*=0.44; refer to Table 6 for full model details). Together, these factors explained 61.7% of the age-adjusted association of depression with incident PAD events. Formal interaction testing demonstrated that the association between depression and PAD did not differ by age, sex, or ethnicity (*P*<0.05).

**Figure:**
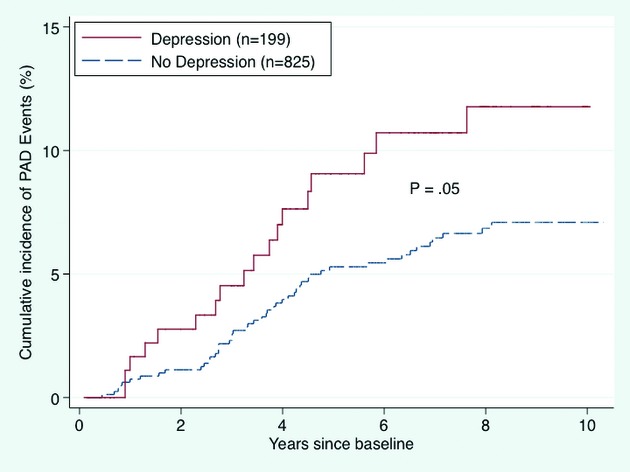
Cumulative incidence of PAD events according to depressive symptoms status. Prospective PAD events by depressive symptoms status. Patients with depressive symptoms were more at risk of developing PAD events during follow-up (*P*=0.05). “No Depression” indicates no depressive symptoms according to a Patient Health Questionnaire score <10; “Depression,” depressive symptoms according to a Patient Health Questionnaire score ≥10. PAD, peripheral artery disease.

**Table 5.** Association of Depression and Incident PAD Events During Follow-Up With Sequential Adjustment for Potential Confounders and Mediators*

Variable	Hazard Ratio (95% CI)	*P*
Adjusted for age and sex only	2.09 (1.09–4.00)	0.03
Adjusted for age, sex, and race/ethnicity	1.93 (0.99–3.74)	0.05
Adjusted age, sex, race/ethnicity, comorbid conditions, and medication use	1.70 (0.87–3.35)	0.12
Adjusted for age, sex, race/ethnicity, and PAD risk factors	1.64 (0.82–3.26)	0.16
Adjusted for age, sex, race/ethnicity, and inflammation	1.75 (0.90–3.39)	0.10
Adjusted for age, sex, race/ethnicity, and health behaviors	1.62 (0.82–3.21)	0.17
Adjusted for age, sex, race/ethnicity, comorbid conditions, medication use, PAD risk factors, inflammation, and health behaviors	1.33 (0.65–2.71)	0.44

Adjusted associations between depression and incident PAD events were estimated by using Cox models with sequential addition of candidate mediators in participants without history of PAD (n=951). Patients with a history of PAD at baseline were excluded from this analysis. PAD, peripheral artery disease.

* Included all variables that changed the effect size between depression and PAD by >5%: demographics (age, race), comorbid conditions and medication use (history of congestive heart failure, diabetes, history of PAD, statins and diuretics), PAD risk factors (high-density lipoprotein, triglycerides, serum creatinine), inflammation (log IL-6), and health behaviors (smoking, physical inactivity).

**Table 6.** Full Model for Incident PAD Events During Follow-Up

Variable	Hazard Ratio (95% CI)	*P*
Depression	1.33 (0.65–2.71)	0.44
Age (per 5 y)	1.28 (1.09–1.51)	0.003
Male	1.57 (0.59–4.15)	0.36
Race/ethnicity		
White	1.00 (ref)	Ref
Hispanic	0.78 (0.23–2.64)	0.69
Asian	0.52 (0.15–1.80)	0.30
African American	0.82 (0.34–1.97)	0.65
Other	2.13 (0.71–6.43)	0.18
Congestive heart failure	1.59 (0.80–3.18)	0.19
Diabetes	1.16 (0.60–2.25)	0.65
Statins	1.81 (0.87–3.78)	0.12
Diuretics	1.78 (0.94–3.38)	0.08
High-density lipoprotein (per 5-mg/dL decrease)	0.99 (0.87–1.13)	0.92
Triglyceride (per natural log increase)	1.54 (0.91–2.60)	0.11
Creatinine (per 0.1 mg/dL)	1.04 (1.01–1.06)	0.005
IL-6 (per natural log increase)	2.02 (1.25–3.24)	0.004
Smoking		
Never	1.00 (ref)	ref
Former	1.57 (0.70–3.50)	0.27
Current	5.07 (1.99–12.9)	0.001
Physical inactivity	1.03 (0.85–1.25)	0.73

Adjusted associations between risk factors including depression and incident PAD events were estimated by using Cox models in participants without history of PAD (n=951). Patients with a history of PAD at baseline were excluded from this analysis. PAD, peripheral artery disease; IL, interleukin.

## Discussion

In this large cohort of patients with CAD, we found that depressive symptoms were significantly associated with prevalent PAD at baseline and with the occurrence of incident PAD events. This association was explained partially by modifiable risk factors such as smoking and lack of physical activity; comorbid conditions such as diabetes mellitus and lipid abnormalities; and inflammation. This information will be important for patients and providers as they try to prevent the development and progression of PAD. These findings suggest that more aggressive treatment of these risk factors could reduce the excess risk of PAD associated with depression.

### Depression and PAD: A Rising Challenge

An emerging body of literature has suggested that depression is associated with greater risk of PAD and worse functional and postoperative outcomes among patients with PAD. Still, the mechanisms of this association are unknown. Wattanakit and colleagues^19^ demonstrated in the Atherosclerosis Risk in Communities Study that psychological factors, including depression, were associated with greater incident PAD. Arseven and colleagues^44^ reported that patients with PAD had twice the risk of comorbid depression than control patients without PAD, though this association was not significant after adjustment for age, education, and other comorbidities. The results of our study are consistent with their findings in view of the fact that adjustment for other risk factors attenuated the strength of the association between depression and PAD. Furthermore, Wong and colleagues,^45^ using data from an elderly Asian population, demonstrated that depressive symptoms were associated with PAD after adjustment for stroke and other cardiovascular diseases. The authors commented that more prospective data were needed to better understand the links between depression and PAD. Our study expands on this existing literature by demonstrating that confounding by modifiable cardiovascular risk factors is partly responsible for this association.

In addition to increasing the risk of PAD, depression also appears to affect the functional status and symptoms of patients with PAD. McDermott and colleagues^46^ found that depression was present in 22% of patients with PAD and that depressive symptoms were associated with greater impairment in lower-extremity functioning. Other studies have demonstrated that, among patients with PAD, those with greater depressive symptoms have more dramatic annual declines in functional performance,^20^ reduced walking distance,^21^ and reduced quality-of-life benefit after revascularization.^22^ Thus, depression can impair health and quality of life in patients even after PAD treatment. Cherr and colleagues^24^ also demonstrated that depression is associated with cardiovascular events and progression of contralateral PAD in patients undergoing lower-extremity revascularization. Furthermore, patients with depression had worse primary assisted and secondary patency and increased risk of recurrent symptomatic PAD.^23^ Other studies have confirmed that patients with depression do worse after surgery for PAD, including having less functional improvement after endovascular procedures^22^ and greater need for revascularization.^47^

It is important to contemplate another aspect of the association between depression and PAD: the direction of the association. As reported by McDermott and colleagues, it is difficult to determine if greater depressive symptoms precede functional walking impairment or vice versa.^46^ On the basis of the present study, we can state that in a cross-sectional design, an association between depression and PAD exists. In the longitudinal design of this study, patients with depressive symptoms were more likely to experience PAD events. It is plausible that patients experiencing functional impairment in their walking capacity secondary to PAD develop depressive symptoms. These depressive symptoms could then further contribute to a decline in their functional status and could precipitate a vicious circle of worsening health behaviors and increased inflammation, leading to more PAD events. An alternative explanation is that depression in itself leads to an increase in modifiable risk factors such as smoking and physical inactivity, which then lead to an increased risk of PAD. One should remember that these hypotheses are highly speculative at the present time and cannot be confirmed with the present data.

### Potential Mediators

Our findings and prior research demonstrate that depression is an important risk factor for the development of PAD, which suggests that interventions to prevent or treat depression could have secondary benefits in reducing PAD risk. An important next step is to understand the mechanisms by which depression might increase PAD risk, with a view toward identifying interventions that might block these causal pathways. After accounting for confounding of depression by demographics, comorbid conditions, and traditional PAD risk factors, we found evidence that inflammation and health behaviors could mediate the effects of depression on PAD. Inflammation is important, given that a majority of patients with major depression develop an increased state of inflammation within a context of chronic psychosocial stress and that they demonstrate an activation of the inflammatory response, including an increased number of peripheral leukocytes (monocytes and neutrophils)^48^ as well as an increase in acute-phase proteins.^49^ Some reports have demonstrated that psychological stress leads to an increase in IL-6, CRP, and nuclear factor-KB.^50–51^ Furthermore, depression itself seems to lead to a state of chronic low-grade inflammation.^52–53^ In our study, depressed patients had higher levels of inflammation, but adjustment for inflammation did not explain all of the effect of depression on PAD. An additional large portion of the association between depression and PAD seemed to be explained by poor health behaviors, particularly smoking and physical inactivity, which is consistent with findings in patients with CAD.^17,33^ After adjustment for confounders, inflammation, and health behaviors, there was no significant association between depression and PAD.

### Implications for Clinical Practice

The findings of this study have important implications for clinical practice. Depressive symptoms seem to be strongly associated with PAD, and this link is explained largely by traditional cardiovascular risk factors. This suggests that depressive symptoms could be a risk factor for PAD through alterations in health behaviors, inflammation, and known risk factors for PAD. Because there is rising evidence that treatment of depression can improve certain risk factors for cardiovascular disease,^54–56^ perhaps an argument could be made that it would be worthwhile to treat depression (even if the link is primarily via other risk factors) so as to reduce risk of PAD. It also should be acknowledged that there are, of course, many reasons to treat depression. Hence, this study highlights the need for providers to have a high index of suspicion for depression in the setting of PAD. Healthcare professionals treating PAD can play an important role in prevention and treatment of PAD in the setting of depression by encouraging their patients with depressive symptoms to get treatment and by underscoring the association between depression and PAD progression, function, and quality of life, as well as by management of the risk factors associated with PAD. Lastly, the present study further outlines the importance of screening for traditional risk factors in the PAD population and of properly addressing these risk factors through lifestyle modification and/or medical treatment.

### Limitations

Our study findings must be interpreted in light of several limitations. First, the Heart and Soul Study includes mostly urban men with existing heart disease. Although this might limit the generalizability of results, this population is an important one to study because of their high risk for development of PAD and for recurrent PAD events. Second, neither the baseline diagnosis of PAD determined by self-report (prevalent PAD) nor prospective PAD events were confirmed by ankle–brachial indices, possibly resulting in underdocumentation of the true prevalence of PAD in this cohort and limiting the interpretation of the results. It is also possible that with the self-report questionnaire, individuals might overreport PAD when the actual pathology is another lower-extremity disorder, such as a deep vein thrombosis, varicose veins, osteoarthritis, or spinal stenosis. Nevertheless, the questionnaire given to patients distinctly identified other lower-extremity pathologies. Furthermore, our findings remained consistent when carefully adjudicated, prospective events were assessed, strengthening the evidence for an association of depression and PAD. Our power to assess the association of depression and incident PAD events was limited by the number of outcomes, and additional follow-up of the cohort could improve power for these analyses. With regard to the fact that patients with depressive symptoms were less likely to be on statins, the difference in statin use in itself could increase the risk of developing PAD, which should be considered another limitation of this study. Finally, we did not collect information on indices of PAD severity such as the ankle–brachial index, so we could not adjust for this in our analyses. Prior research, however, demonstrated that objective disease indicators such as the ankle–brachial index are associated only modestly with depression in PAD.^22^

## Conclusions

We found that depressive symptoms were associated with greater self-reported history of PAD at baseline and greater risk of prospective PAD events over 7 years of follow-up. These data add to a growing number of studies demonstrating that depression is an important risk factor for the development of PAD. Furthermore, because elevations in traditional, modifiable cardiovascular risk factors partially explain the excess risk of PAD associated with depression, the identification of depression in PAD needs to be prioritized, and accompanying risk factors should be assessed and aggressively treated. In view of the continued high morbidity and mortality rates associated with PAD, the need for adjunctive modalities in the prevention and treatment of PAD remains essential.
